# Impact of Body Mass Index on Clinical Outcomes in Myocardial Infarction Patients Undergoing Coronary Stenting with Dual Antiplatelet Therapy

**DOI:** 10.3390/biomedicines13112792

**Published:** 2025-11-16

**Authors:** Réka Aliz Lukács, Dániel Tornyos, András Jánosi, András Komócsi

**Affiliations:** 1 Heart Institute, Medical School, University of Pécs, Ifjúság útja 13, H-7624 Pécs, Hungary; 2 Gottsegen National Institute of Cardiology, Haller Street 29, H-1096 Budapest, Hungary; andras.janosi@gokvi.hu

**Keywords:** body mass index, coronary intervention, dual antiplatelet therapy, clopidogrel, prasugrel, decision curve analysis, Hungarian Myocardial Infarction Registry, acute coronary syndromes

## Abstract

**Background**: Dual antiplatelet therapy (DAPT), combining aspirin with a P2Y12 receptor inhibitor (P2Y12i), remains central to the management of acute myocardial infarction (MI), especially in patients undergoing percutaneous coronary intervention (PCI). However, the pharmacodynamic response to antiplatelet therapy may vary with body composition. This study investigates the association between body mass index (BMI) and clinical outcomes in MI patients treated with PCI and DAPT. **Methods**: This retrospective cohort study analyzed data from 52,119 MI patients treated with coronary stenting from 2014 to 2021, sourced from the Hungarian Myocardial Infarction Registry. Patients were stratified into clopidogrel-based (*n* = 44,480) and potent P2Y12i-based (prasugrel or ticagrelor; *n* = 7639) DAPT cohorts. Clinical outcomes—including 12-month mortality and ischemic events—were assessed across BMI categories. Kaplan–Meier analysis and LASSO Cox regression identified predictors of mortality, while decision curve analysis (DCA) evaluated the net clinical benefit of potent P2Y12i across BMI strata. **Results**: Univariate and multivariate Cox regression analyses identified BMI and potent P2Y12i treatment as significant predictors of 365-day mortality, with higher BMI associated with lower observed rates of mortality, major adverse cardiovascular events (MACEs), and stroke. However, higher BMI was also associated with an increased risk of repeat revascularization and PCI. This study found that the protective effect of potent P2Y12i treatment was consistent across different BMI categories. **Conclusions**: In patients with MI undergoing PCI, elevated BMI was paradoxically associated with more favorable short-term outcomes, including reduced mortality. Potent P2Y12i therapy demonstrated a consistent benefit across BMI categories, supporting its broad application irrespective of body mass.

## 1. Introduction

Timely coronary revascularization and dual antiplatelet therapy (DAPT), combining aspirin and an ADP P2Y12 inhibitor (P2Y12i), remain the cornerstone of treatment for acute coronary syndrome (ACS). Potent P2Y12 inhibitors, prasugrel and ticagrelor, have demonstrated more consistent and effective platelet inhibition than clopidogrel, the former gold-standard therapy [1]. However, the significant reduction in ischemic events with these drugs came with an increased risk of bleeding [1,2].

Importantly, body weight influences both the efficacy and safety of P2Y12i therapy. Patients with lower weight exhibit higher levels of active thienopyridine metabolites, resulting in enhanced platelet inhibition, lower residual platelet reactivity and increased bleeding risk [3]. This pharmacokinetic association prompted a reduction in the recommended prasugrel dose for patients weighing less than 60 kg [4]. However, compared to weight, body mass index (BMI) may offer a more robust estimate of drug exposure, as it accounts for body weight and height, and it is a well-established marker of cardiovascular thrombotic and bleeding risk. Accordingly, BMI may also serve as a more precise surrogate to evaluate the clinical impact of antiplatelet regimens [5].

We aimed to characterize the association between baseline BMI and 12-month outcomes after PCI for myocardial infarction in a large, unselected national cohort and to explore how this relationship interacts with contemporary P2Y12 inhibitor therapy. By linking body composition-related risk with treatment intensity in real-world practice, our study provides novel insights into personalized antiplatelet management across the BMI spectrum.

## 2. Methods

### 2.1. Study Design and Patients

#### 2.1.1. Hungarian Myocardial Infarction Registry (HUMIR)

The Hungarian Myocardial Infarction Registry (HUMIR) is a comprehensive, prospective, and mandatory national registry designed to collect clinical data on patients treated for acute myocardial infarction (AMI) within Hungary. Since 2014, HUMIR has operated under the legal framework of Hungary’s statute CCXLVI./2013 and it was integrated into the ESC EuroHeart program in 2021 [6]. Previous publications have provided detailed accounts of the methods used for data collection and patient follow-up [7,8,9]. The HUMIR operates as an extensive data repository, capturing a broad range of variables from demographics to procedural characteristics related to coronary interventions [10,11]. Data collection is conducted with approval from the central ethics committee, ensuring that all patient-level information is anonymized to preserve confidentiality while enabling robust clinical analyses.

#### 2.1.2. Patient Selection and Clinical Endpoints

This study included patients hospitalized with AMI who underwent PCI during the acute phase and were initiated on DAPT, consisting of low-dose aspirin (ASA) and a P2Y12 inhibitor (clopidogrel, prasugrel, or ticagrelor). The choice of P2Y12i was at the discretion of the treating physician. Patients were stratified by BMI into four categories, underweight, normal weight, overweight, and obese, according to the World Health Organization.

The primary endpoints of interest were all-cause mortality, recurrent myocardial infarction, and stroke. Major adverse cardiovascular events (MACEs) were defined as the composite of mortality, repeated myocardial infarction, or stroke. These events were recorded through linkage with the National Health Insurance Fund (NHIF) database. All primary endpoints were assessed within the first 12 months following the index PCI. Covariate selection was based on established clinical relevance to outcomes after PCI. The multivariable models were adjusted for age, sex, smoking status, diabetes, hypertension, prior myocardial infarction, prior stroke, heart failure, chronic kidney disease, and STEMI presentation.

### 2.2. Statistical Analysis

Baseline characteristics were summarized using descriptive statistics. Continuous variables were reported as means with standard deviations (SDs) depending on distribution, and categorical variables were presented as frequencies and percentages. A descriptive overview of BMI distribution is shown in Supplementary Figure S1.

Age was analyzed as a continuous covariate, with hazard ratios expressed per 10-year increase. In a sensitivity analysis, age groups (≤55, 56–65, 66–75, >75 years) were used to test for a BMI × age interaction.

Given significant differences in baseline characteristics between treatment groups, propensity score matching (PSM) was performed to reduce confounding. The PS model included age, sex, BMI, blood pressure, heart rate, diabetes, hypertension, a history of cardiovascular disease, anemia, renal dysfunction, and STEMI diagnosis. Covariate balance after matching was assessed using standardized mean differences, with values < 0.1 considered acceptable. To minimize potential collider bias, BMI was modeled as a baseline continuous covariate alongside major determinants of both obesity and outcomes (age, diabetes, hypertension, heart failure, and renal dysfunction).

Kaplan–Meier survival estimates with log-rank tests were used to compare mortality and MACEs across BMI categories. Cox proportional hazards models were employed to assess associations between BMI and clinical outcomes. Differences in endpoints were analyzed using univariate Cox regression, while multivariate models with interaction terms were used to evaluate potential effect modification by BMI. BMI was modeled both as a continuous and categorical variable.

For multivariate analysis, Least Absolute Shrinkage and Selection Operator (LASSO) Cox regression was applied to identify the most robust predictors of 365-day mortality while minimizing model overfitting. The optimal lambda value was selected through 10-fold cross-validation to optimize model performance and predictive accuracy (Supplementary Figure S2), and the final model coefficients were extracted and reported. The cross-validation curve was plotted to illustrate the selection process of the optimal lambda value. Significant predictors were visualized with corresponding hazard ratios (HRs) and 95% confidence intervals (CIs) to facilitate clinical interpretation.

Temporal stability of the association between BMI and clinical outcomes was evaluated by fitting bivariate Cox regression models stratified by admission year (2014–2021), including BMI (per 5 kg/m^2^ increase) and P2Y12 inhibitor treatment as covariates. We also examined BMI × year and BMI × P2Y12 interaction terms to assess potential effect modification over time. The results were summarized as yearly hazard ratios (HRs) with 95% confidence intervals and are presented in Supplementary Table S1 and Figure S3.

### 2.3. Bivariate Regression Analysis

To assess the impact of P2Y12i treatment (clopidogrel versus potent P2Y12 inhibitors) and BMI on clinical endpoints, bivariate Cox regression was used for time-to-event outcomes and logistic regression for binary outcomes. For each endpoint, two models were fitted: one with the treatment variable and one with BMI as a continuous predictor, scaled per 5-unit increase. To evaluate potential effect modification by BMI, interaction terms between treatment and BMI were included in the regression models. The significance of the interaction was assessed via the *p* value of the interaction term.

Kaplan–Meier plots were constructed to illustrate outcome risks across BMI categories. Restricted cubic spline Cox regression models were used to assess nonlinear interactions between BMI and treatment. Hazard ratios with 95% CIs were computed, and visualizations included treatment-specific confidence intervals and WHO BMI category thresholds.

### 2.4. Decision Curve Analysis

Decision curve analysis (DCA) was performed to evaluate the net clinical benefit of potent P2Y12i treatment in reducing 12-month mortality and MACEs. Predicted risks were obtained from Cox proportional hazards models, treating BMI as a continuous variable. Interaction terms between treatment and age were also assessed to evaluate age-related heterogeneity in treatment response. Predicted risks were used to construct data frames containing binary outcomes and corresponding probabilities. DCA was performed across threshold probabilities ranging from 0 to 1 (in 0.01 increments), and net benefit curves were plotted [12]. All statistical analyses were conducted using R version 4.2.2.

## 3. Results

**Figure 1:**
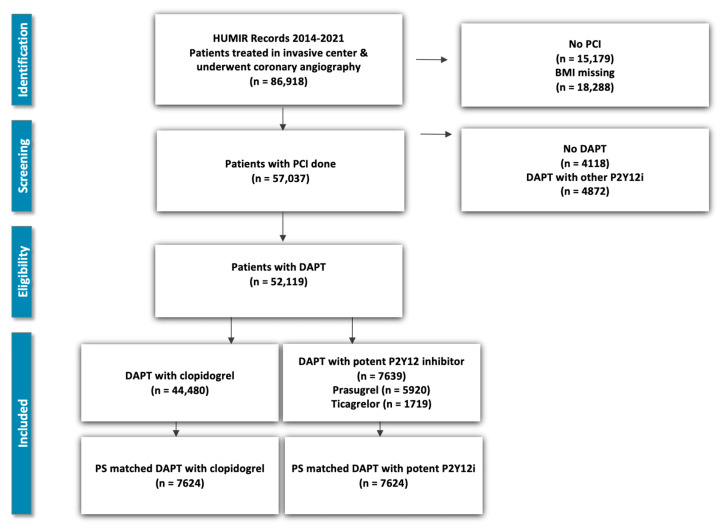
**Patient Selection Flowchart**. A flow diagram illustrating the extraction of eligible cases from the Hungarian Myocardial Infarction Registry (HUMIR), restricted to patients with AMI treated with PCI and receiving DAPT (aspirin plus clopidogrel or a potent P2Y12 inhibitor).

A total of 52,119 patients were included in this study, comprising 44,480 patients treated with clopidogrel-based dual antiplatelet therapy (DAPT) and 7639 patients treated with a potent P2Y12 inhibitor (prasugrel or ticagrelor). The patient selection process is summarized in Figure 1.

### 3.1. Baseline Characteristics

Baseline characteristics of the study population are presented in Table 1. In the unmatched cohort, numerous differences were observed between treatment groups. Patients in the potent P2Y12i group were younger, more often male, and had higher BMI compared to those receiving clopidogrel. They also more frequently presented with ST elevation myocardial infarction (STEMI) and had a higher prevalence of diabetes and hypertension, while prior AMI was less common.

Notably, systolic and diastolic blood pressures and heart rate at presentation were slightly but significantly higher in the potent P2Y12i group. The prevalence of severe anemia and prior stroke was lower and mean hemoglobin levels were higher in the potent P2Y12i cohort (Table 1). To minimize confounding, propensity score matching was performed, resulting in a balanced cohort of 15,248 patients (7624 in each group). Post-matching comparisons showed no significant differences in baseline characteristics, confirming the effectiveness of the matching process.

### 3.2. Univariate and Multivariate Cox Regression Analyses

Univariate Cox regression assessed associations between 1-year all-cause mortality and demographic, clinical, and procedural variables. Analyses were performed in both the full cohort (*n* = 52,119) and the matched cohort (*n* = 15,248), with the results shown in Table 2.

Treatment with a potent P2Y12 inhibitor and having higher BMI (per 5 kg/m^2^) were associated with reduced mortality in both cohorts. Compared to normal weight (BMI 18.5–24.9), overweight and obese categories showed lower mortality, while underweight was linked to a higher mortality risk only in the full cohort but this effect did not reach the level of statistical significance after PS matching.

Additional significant predictors of higher mortality in both populations included female sex, a history of MI, HF, hypertension, stroke, diabetes, PAD, prior PCI, and CABG. Age independently predicted mortality (HR 1.8, 95% CI 1.7–1.9 per 10 years). The inverse BMI–mortality association was consistent across age groups (*p* for interaction = 0.42). Paradoxically smoking and presentations as STEMI were associated with lower mortality risk.

In the multivariate Cox regression analysis, adjusting for other covariates, BMI and P2Y12i treatment prevailed as significant predictors of 365-day mortality. The protective effect of each 5-unit increase in BMI persisted (HR 0.8, 95% CI, 0.8–0.8; *p* < 0.001). Similarly, the protective effect of having BMI of 25–29.9 was maintained (HR 0.7, 95% CI, 0.6–0.7), with higher BMI categories also showing consistent protective effects (Supplementary Figure S2).

**Figure 2:**
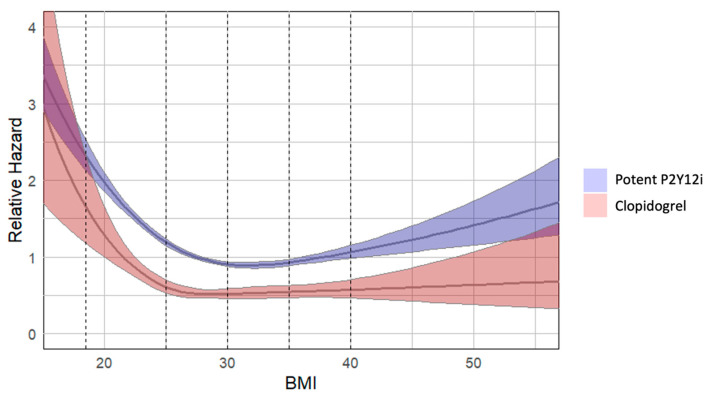
**Relative hazard of 365-day mortality by BMI and treatment group**. The figure illustrates the relative hazard of 365-day mortality as a function of BMI for two treatment groups: control (blue) and treatment (red). The solid lines represent the estimated hazard ratios derived from a Cox proportional hazards model with restricted cubic splines, while the shaded areas indicate the 95% confidence intervals. Vertical dashed lines mark the World Health Organization (WHO) BMI categories: underweight (<18.5), normal weight (18.5–24.9), overweight (25–29.9), obesity class I (30–34.9), obesity class II (35–39.9), and obesity class III (≥40).

Higher baseline BMI was inversely associated with 12-month mortality (adjusted HR ~0.85 per 5 kg/m^2^). The relationship was nonlinear, with a gradual risk decrease up to approximately 30 kg/m^2^ and flattening at higher levels. This pattern is illustrated by the restricted cubic spline curve (Figure 2).

The inverse association between BMI and 1-year mortality was consistent throughout the study period. Year-stratified analyses showed comparable hazard ratios for BMI across 2014–2021, with no significant BMI × year interaction (*p* > 0.10 for all years). Kaplan–Meier curves (Supplementary Figure S3) demonstrated early and sustained separation among BMI categories, and yearly analyses of a BMI × P2Y12 interaction revealed no major heterogeneity, confirming the temporal stability of both BMI- and treatment-related effects.

### 3.3. Bivariate Regression Analyses

To further explore endpoint-specific effects, bivariate regression models assessed the impact of P2Y12 treatment and BMI on mortality, MACEs, stroke, MI, and revascularization. The full results are summarized in Table 3. BMI was consistently protective against mortality (HR: 0.82; 95% CI: 0.80–0.84), MACEs (HR: 0.87; 95% CI: 0.85–0.89), and stroke (HR: 0.87; 95% CI: 0.81–0.93). No significant effect modification by BMI was observed for any of these endpoints, as interaction *p* values were all >0.20.

Higher BMI was linked to an increased risk of repeat PCI [13], underscoring the complexity of BMI’s role in cardiovascular outcomes. BMI had no significant association with MI or CABG incidence.

### 3.4. Decision Curve Analysis

**Figure 3:**
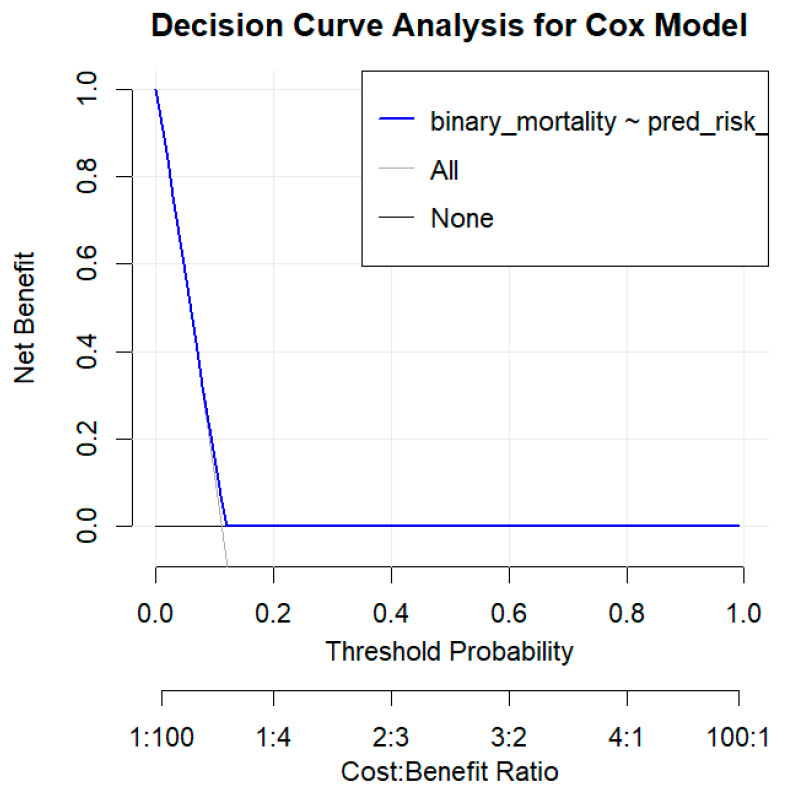
**Decision Curve Analysis for BMI-based Mortality Risk Prediction in the Use of Potent P2Y12 Inhibitors vs. Clopidogrel.** The decision curve analysis (DCA) illustrates the net benefit of using a BMI-based model to decide between treating patients with potent P2Y12 inhibitors or clopidogrel based on predicted mortality risk. The blue line represents the net benefit of the BMI-based decision model. The black lines represent the net benefit of treating all patients with potent P2Y12 inhibitors (“All”) and treating none (“None”).

Decision curve analysis (DCA) assessed the clinical utility of using BMI to guide P2Y12i treatment decisions. Decision curve analysis (DCA) showed a marginal or limited net benefit of BMI-based models in mortality risk prediction, illustrating that BMI alone provides modest clinical discrimination (Figure 3).

## 4. Discussion

There are limited data regarding the prognostic implications of BMI across a full spectrum of antiplatelet regimens after PCI. We aimed to integrate potent P2Y12 inhibitor use into the assessment of the obesity paradox. This approach extends prior work focused solely on metabolic risk or platelet reactivity by linking body composition with pharmacologic treatment patterns and outcomes. In this large nationwide cohort of MI patients undergoing PCI, higher BMI was paradoxically associated with more favorable 12-month outcomes, including significantly lower all-cause mortality and MACE rates, compared to normal-weight patients [14,15]. These findings align with a substantial body of research reporting improved short-term prognosis in overweight and moderately obese cardiovascular patients. Meta-analyses and registries have shown a U- or J-shaped relationship between BMI and outcomes in coronary artery disease (CAD), with underweight patients experiencing the highest risks and overweight patients often faring better than normal-weight individuals [13,14,16]. For example, Romero-Corral et al. demonstrated in a systematic review that overweight and mildly obese CAD patients had lower mortality than their leaner counterparts [17]. Importantly, our data confirm that lower BMI is not independently predictive of better outcomes in the modern MI population—if anything, underweight patients tended to have higher (unadjusted) mortality—highlighting that traditional risk calculations should not assume that leaner is always healthier in the context of acute cardiac events. While our findings illustrate the so-called “obesity paradox” [16,18,19] in acute myocardial infarction, this likely reflects the complex interplay of body composition, metabolic reserve, and comorbidity burden rather than a protective effect of excess adiposity per se. Importantly, these results should not be interpreted as opposing the well-established benefits of intentional, metabolically driven weight reduction—particularly with GLP-1 receptor agonists—which have been shown to improve long-term cardiometabolic and survival outcomes.

A pattern resembling the well-known “smoker’s paradox” was observed in unadjusted analyses, with lower crude mortality among current smokers; however, this association disappeared after multivariable adjustment. These findings suggest that the apparent paradox likely reflects confounding by younger age and lower comorbidity burden rather than a genuine protective effect of smoking.

Our study also underscores the prognostic importance of DAPT choice and supports the broad use of potent P2Y12 inhibitors in MI patients irrespective of BMI. Consistent with prior randomized trials, we found that ticagrelor- or prasugrel-based DAPT was associated with substantially lower mortality and ischemic events compared to clopidogrel-based therapy [20]. In TRITON-TIMI 38, a pivotal trial of prasugrel vs. clopidogrel in ACS, prasugrel reduced the composite risk of cardiovascular death, MI, or stroke relative to clopidogrel, albeit with an increased incidence of major bleeding (2.4% vs. 1.8%). Notably, that study identified subgroups at particular risk of bleeding complications on prasugrel—namely patients with a history of TIA or stroke, those ≥ 75 years old, and those with low body weight (<60 kg) [21]. As a result, current guidelines contraindicate prasugrel in patients with prior stroke/TIA and recommend a reduced maintenance dose (5 mg daily) for individuals weighing < 60 kg (as well as for the elderly ≥ 75 years) [22]. Pharmacokinetic analyses have confirmed that low-body-weight patients experience ~30% higher exposure to active prasugrel metabolites, which likely explains their heightened bleeding risk [23]. Our real-world data reflect these principles: patients at the extremes of low BMI (who often overlap with low body weight) derive less absolute benefit from potent P2Y12 inhibitors, presumably due to an increased bleeding hazard, whereas the survival benefit of potent agents is pronounced in those with BMI in the normal to obese range. In contrast, ticagrelor (as studied in the PLATO trial) does not require weight-based dose adjustment and demonstrated superior efficacy over clopidogrel across a broad spectrum of ACS patients, including significant reductions in MI and cardiovascular mortality. Our findings are in line with PLATO and the ISAR-REACT5 trials, confirming that more potent platelet inhibition translates into better ischemic outcomes in PCI-treated MI patients, without evidence of attenuation in efficacy among higher-BMI individuals [24,25]. Our findings are consistent with recent data from the DAPT-TR study (Öz et al., 2024), which also demonstrated the prognostic relevance of antiplatelet intensity across patient subgroups [26]. In an earlier report from the HUMIR analysis, potent P2Y12 inhibitors significantly reduced 1-year mortality compared with clopidogrel across most age groups, with consistent benefit even in elderly patients (HR 0.82 for 75–79 years; HR 0.79 for ≥80 years). Importantly, although age contributed to overall risk prediction, treatment effects did not differ significantly by age, supporting the uniform effectiveness of potent P2Y12 inhibitors across age categories [27].

Current clinical guidelines from both Europe and the US reflect the advantages of potent P2Y12 inhibitors and the need to tailor therapy to patient risk profiles. The 2017 ESC focused update on DAPT (in collaboration with EACTS) recommends 12 months of DAPT after ACS-PCI and gives preference to ticagrelor or prasugrel over clopidogrel for most patients, provided there are no contraindications [1,28]. Similarly, ACC/AHA guidelines for ACS management advocate using ticagrelor or prasugrel in PCI-treated patients in place of clopidogrel due to their superior efficacy in preventing major adverse events [29]. Our results reinforce these recommendations, showing a robust mortality reduction with potent agents in routine practice. At the same time, guidelines urge caution and dose adjustments for certain subpopulations: for example, the ACC/AHA and ESC guidelines concur that prasugrel’s maintenance dose should be lowered to 5 mg in patients under 60 kg (and generally avoided or used with caution in the very elderly). In our cohort, physicians’ discretion likely reflected these precautions, and the consistency of benefit from potent P2Y12 therapy across BMI groups suggests that when used appropriately, ticagrelor and prasugrel improve outcomes irrespective of body size. Of note, we did not observe any signal of diminished efficacy of potent P2Y12 inhibitors in obese patients. This is reassuring given concerns that fixed dosing of antiplatelet drugs might be less effective in individuals with a high body mass (due to a larger volume of distribution or other obesity-related pharmacokinetic changes) [3]. In fact, the absence of a BMI–treatment interaction (interaction *p* > 0.20 for mortality and MACEs in our analysis) indicates that the relative benefit of potent DAPT was uniform from lean to obese. A prespecified sub-analysis of the GLOBAL LEADERS trial supports this finding: BMI did not significantly influence the comparative outcomes of an experimental ticagrelor-based strategy versus standard DAPT [20]. Thus, our data and prior studies suggest that clinicians should not withhold potent P2Y12 inhibitors from overweight or obese MI patients out of efficacy concerns; these patients remain excellent candidates for intensive antiplatelet therapy, provided their bleeding risk is acceptable. On the other end of the spectrum, patients with very low BMI (e.g., BMI < 18–20) may warrant special consideration. In our spline analysis, the mortality benefit of potent P2Y12 therapy became apparent only above ~19 kg/m^2^, hinting that extremely low-BMI patients (who are often frail or malnourished) gain less net benefit. Such individuals are more prone to bleeding complications and adverse outcomes generally, which may offset the ischemic protection conferred by more potent P2Y12 inhibition. This nuanced finding underscores the importance of individualized therapy—as reflected in guidelines that classify advanced age and low body weight as factors favoring a more conservative DAPT approach (e.g., using clopidogrel or shortened DAPT duration in high-bleeding-risk scenarios) [28]. Our results, taken together with the literature, support a strategy of using potent P2Y12 inhibitors broadly in post-PCI patients to maximize efficacy while applying dose adjustments or de-escalation strategies in those at elevated bleeding risk (such as very-low-BMI or frail patients). Notably, the TROPICAL-ACS trial demonstrated that guided de-escalation from prasugrel to clopidogrel based on platelet function testing can be non-inferior to 12-month prasugrel in ACS patients, and a subgroup analysis from that trial suggested patients without overweight (BMI < 25) may derive particular benefit from de-escalation due to their higher bleeding liability [3]. These data align with our findings and highlight that “one size fits all” may not apply to DAPT intensity—especially at the extremes of body weight—even though the overall superiority of potent P2Y12 agents is maintained across the population.

While BMI is associated with outcomes, it does not capture enough prognostic or hemorrhagic risk information to guide DAPT intensity on its own. Therefore, clinical judgment and comprehensive risk scores (incorporating age, comorbidities, prior bleeding, etc.) should remain the basis for DAPT decisions, rather than simplistic cut-offs based on body mass. Indeed, recent guidelines emphasize balancing ischemic and bleeding risk when choosing DAPT regimens, using validated risk scores (e.g., GRACE, CRUSADE) and clinical factors—a nuanced process that cannot be reduced to BMI criteria. Our use of DCA, in line with contemporary recommendations for evaluating prediction models, highlights that adding BMI to a decision algorithm for P2Y12 selection does not meaningfully improve net clinical benefit beyond the default strategy of potent DAPT for all suitable patients. The DCA was intended as an exploratory illustration of BMI’s limited incremental value for clinical risk stratification rather than as evidence of clinical applicability. In the future, more sophisticated risk prediction tools (potentially combining BMI with other variables such as platelet function or genetic polymorphisms) might be developed to personalize antiplatelet therapy. However, BMI should be viewed as just one piece of the risk puzzle; our findings may help inform more individualized antiplatelet strategies, particularly in patient subgroups where body composition could influence both pharmacodynamics and clinical risk.

## 5. Conclusions

In summary, our nationwide analysis confirms an obesity paradox in contemporary MI patients undergoing PCI: higher BMI is associated with lower 1-year mortality and MACEs, despite a higher incidence of repeat revascularization. Meanwhile, the use of potent P2Y12 inhibitors (ticagrelor or prasugrel) confers a mortality benefit uniformly across BMI categories, supporting their routine use in ACS patients regardless of body size. Extreme low body weight/BMI may attenuate the net benefit of intensive antiplatelet therapy due to bleeding risk, suggesting a role for individualized de-escalation or dose adjustment strategies in that subset. Current ESC and ACC/AHA guidelines appropriately reflect these nuances, endorsing potent P2Y12 agents for most post-PCI patients while recommending caution in those with low weight or a high bleeding risk. Finally, BMI alone appears insufficient to guide antiplatelet treatment decisions, as evidenced by decision curve analysis, underscoring that a multifactorial risk assessment remains essential for optimizing DAPT in clinical practice. Our findings contribute to the evidence base for personalized antiplatelet therapy and highlight the need for further research into the biological mechanisms behind the obesity paradox in cardiovascular disease.

## 6. Limitations

As this was an observational registry analysis, the possibility of residual or unmeasured confounding cannot be excluded despite comprehensive multivariable and propensity-based adjustments. Therefore, the associations reported should be interpreted as non-causal and hypothesis-generating. BMI was the only systematically collected anthropometric parameter in the registry and therefore served as the basis for our analysis. We acknowledge that BMI does not differentiate between fat and lean mass or capture visceral adiposity, which may have contributed to residual confounding in the observed associations. BMI was assessed only at baseline, and weight trajectories after PCI were not captured; therefore, associations reflect baseline rather than post-procedural obesity status. The registry lacked information on lifestyle factors, physical activity, frailty, nutritional status, and medication adherence, as well as GLP-1/GIP agonist use, which may have influenced outcomes and contributed to residual confounding. Although we formally tested interactions by calendar year, age, and P2Y12 regimen, residual heterogeneity may remain. The registry captured only in-hospital bleeding events, and long-term post-discharge bleeding could not be evaluated, which limited the interpretability of bleeding-related outcomes.

## Figures and Tables

**Table 1:** The baseline characteristics of the patient population before and after propensity score matching.

Clinical Characteristics	Entire Cohort (*n* = 52,119)			Propensity-Matched Cohort (*n* = 15,248)		
	**Clopidogrel**	**Potent P2Y12i**	***p*** **Value**	**Clopidogrel**	**Potent P2Y12i**	***p* Value**
***n***	44,480	7639		7624	7624	
**Men**	28,370 (63.8%)	5117 (67.0%)	<0.01	5158 (67.7%)	5109 (67.0%)	0.41
**Age (years)**	65.2 (12.3)	61.4 (10.9)	<0.01	61.3 (11.9)	61.4 (10.9)	0.75
**Weight (kg)**	81.1 (16.9)	86.2 (17.6)	<0.01	86.1 (17.9)	86.2 (17.6)	0.59
**Height (cm)**	169.3 (8.7)	170.4 (8.6)	<0.01	170.5 (8.7)	170.4 (8.6)	0.36
**BMI**	28.2 (5.1)	29.6 (5.5)	<0.01 *	29.6 (5.6)	29.6 (5.5)	1.00 *
**<18.5**	550 (1.2%)	44 (0.6%)	<0.01 **	40 (0.52%)	44 (0.58%)	0.38 **
**18.5–24.9**	11,031 (24.8%)	1290 (16.9%)		1361 (17.85%)	1289 (16.91%)	
**25–29.9**	18,758 (42.2%)	3117 (40.8%)		3030 (39.74%)	3111 (40.81%)	
**30–34.9**	98,514 (22.1%)	2044 (26.8%)		2009 (26.35%)	2038 (26.73%)	
**≥35**	4290 (96%)	1144 (15%)		1184 (15.53%)	1142 (14.98%)	
**SMOKER**	19,139 (43.0%)	3264 (42.7%)	0.63	3206 (42.1%)	3257 (42.7%)	0.41
**STEMI**	23,387 (52.6%)	4507 (59.0%)	<0.01	4481 (58.8%)	4496 (59.0%)	0.82
**KILLIP**	1.1 (0.4)	1.1 (0.4)	0.066 *	1.1 (0.4)	1.1 (0.4)	0.13 *
**I**	40,346 (90.9%)	7006 (91.8%)	0.011 **	6975 (91.5%)	7004 (91.9%)	0.68 **
**II**	2958 (6.7%)	455 (6%)		479 (6.3%)	455 (6.0%)	
**III**	709 (1.6%)	97 (1.3%)		92 (1.2%)	97 (1.3%)	
**IV**	369 (0.8%)	73 (1%)		78 (1.0%)	68 (0.9%)	
**Heart rate (bpm)**	80.0 (17.4)	80.6 (16.6)	0.012	80.7 (17.2)	80.6 (16.6)	0.53
**Systolic blood** **pressure (mmHg)**	135.3 (24.2)	137.9 (24.7)	0.012	137.5 (24.5)	137.9 (24.7)	0.31
**Diastolic blood** **pressure (mmHg)**	79.8 (14.3)	81.1 (14.4)	<0.01	81.0 (14.4)	81.1 (14.4)	0.59
**Prior myocardial** **infarction**	6806 (15.3%)	1084 (14.2%)	0.18	1121 (14.7%)	1081 (14.2%)	0.37
**History of** **heart failure**	4138 (9.3%)	447 (5.9%)	<0.01	426 (5.6%)	446 (5.8%)	0.51
**Hypertension**	34,471 (77.5%)	5974 (78.2%)	<0.01	5987 (78.5%)	5964 (78.2%)	0.67
**Prior stroke**	3232 (7.3%)	242 (3.2%)	<0.01	251 (3.3%)	242 (3.2%)	0.71
**Diabetes mellitus**	12,802 (28.8%)	3918 (51.3%)	0.64	3904 (51.2%)	3913 (51.3%)	0.90
**Peripheral artery** **disease**	4705 (10.6%)	625 (8.2%)	<0.01	636 (8.3%)	624 (8.2%)	0.75
**Prior coronary** **intervention**	6704 (15.1%)	1135 (14.9%)	<0.01	1162 (15.2%)	1133 (14.9%)	0.53
**Prior of coronary bypass operation**	1715 (3.9%)	221 (2.9%)	<0.01	226 (3.0%)	219 (2.9%)	0.77
**Kidney disease**	11,106 (25.0%)	1347 (17.6%)	<0.01	1322 (17.3%)	1343 (17.6%)	0.67

Variables include demographics, anthropometric measures, clinical presentation, comorbidities, and prior cardiovascular history. Abbreviations: STEMI = ST elevation myocardial infarction, BMI = body mass index. * comparison of numerical values, ** categorical comparison.

**Table 2:** Univariate analysis of one-year mortality in the entire cohort and the propensity score-matched cohort.

Variable	Entire Cohort (*n* = 51,959)		Propensity-Matched Cohort (*n* = 15,248)	
	**Hazard Ratio (95% CI)**	***p* Value**	**Hazard Ratio (95% CI)**	***p* Value**
**P2Y12i treatment**	0.5 (0.5–0.6)	<0.001	0.7 (0.6–0.8)	<0.001
**BMI** **(per 5-unit increase)**	0.8 (0.8–0.8)	<0.001	0.9 (0.8–0.9)	<0.001
**<18.5**	2.0 (1.7–2.3)	<0.001	1.4 (0.8–2.5)	0.263
**25–29.9**	0.7 (0.6–0.7)	<0.001	0.7 (0.6–0.8)	<0.001
**30–34.9**	0.6 (0.6–0.7)	<0.001	0.6 (0.5–0.8)	<0.001
**≥35**	0.6 (0.6–0.7)	<0.001	0.7 (0.5–0.8)	<0.001
**Men**	0.8 (0.7–0.8)	<0.001	0.8 (0.7–0.9)	<0.001
**Age** **(per 10-year increase)**	1.9 (1.8–1.9)	<0.001	1.8 (1.7–1.9)	<0.001
**Smoker**	0.6 (0.5–0.6)	<0.001	0.6 (0.5–0.7)	<0.001
**STEMI**	0.8 (0.8–0.9)	<0.001	0.9 (0.8–1.0)	0.025
**Prior myocardial infarction**	1.6 (1.5–1.7)	<0.001	1.5 (1.3–1.7)	<0.001
**History of heart failure**	2.9 (2.7–3.1)	<0.001	2.8 (2.3–3.3)	<0.001
**Hypertension**	1.4 (1.3–1.5)	<0.001	1.3 (1.1–1.5)	<0.001
**Prior stroke**	2.3 (2.2–2.5)	<0.001	2.1 (1.6–2.6)	<0.001
**Diabetes mellitus**	1.6 (1.5–1.7)	<0.001	1.6 (1.4–1.8)	<0.001
**Peripheral artery disease**	2.4 (2.2–2.5)	<0.001	2.5 (2.1–2.9)	<0.001
**Prior coronary intervention**	1.2 (1.1–1.3)	<0.001	1.3 (1.1–1.5)	0.003
**Prior of coronary bypass operation**	1.5 (1.4–1.7)	<0.001	1.5 (1.1–2.0)	0.005

This table presents the hazard ratios (HRs) and 95% confidence intervals (CIs) for one-year mortality associated with various clinical variables, including treatment type and body mass index (BMI) categories, in both the entire patient cohort and the propensity score-matched cohort. The analysis compares the effects of potent P2Y12 inhibitors versus clopidogrel-based dual antiplatelet therapy (DAPT) and examines the impact of different BMI ranges on mortality outcomes.

**Table 3:** Bivariate Cox and linear regression analyses of P2Y12 treatment and BMI on individual and composite endpoints.

Clinical Endpoint	Hazard Ratio Clopidogrel Versus P2Y12i	Hazard Ratio BMI (per 5-Unit Increase)	*p* Value Interaction
Mortality	0.53 (95% CI: 0.48–0.58)	0.82 (95% CI: 0.8–0.84)	0.214
MACEs	0.6 (95% CI: 0.56–0.65)	0.87 (95% CI: 0.85–0.89)	0.217
Myocardial infarction	0.75 (95% CI: 0.67–0.85)	0.99 (95% CI: 0.96–1.03)	0.731
Stroke	0.56 (95% CI: 0.45–0.71)	0.87 (95% CI: 0.81–0.93)	0.270

The table presents the results of bivariate Cox regression and bivariate linear regression analyses assessing the impact of P2Y12 treatment and BMI (per 5-unit increase) on various individual and composite endpoints. The table includes hazard ratios (HRs) for clopidogrel versus potent P2Y12 inhibitors and HRs for BMI, along with their 95% confidence intervals (CIs). Additionally, interaction *p* values are provided to evaluate the potential modification effect of BMI on treatment outcomes. Abbreviations: percutaneous coronary intervention (PCI), coronary artery bypass grafting (CABG), and major adverse cardiovascular events (MACEs).

## Data Availability

The data underlying this article will be shared upon reasonable request to the corresponding author.

## Supplementary Materials

The following supporting information can be downloaded at https://www.mdpi.com/article/10.3390/biomedicines13112792/s1: Figure S1. Combined density and bar plot of BMI distribution with WHO categories. This figure displays a combined density and bar plot illustrating the distribution of body mass index (BMI) values among the study population. The density plot (in blue) represents the smooth distribution of BMI values, while the overlaid bar plot (in gray) shows the count of individuals within specific BMI categories. Vertical dashed red lines mark the World Health Organization (WHO) BMI classification thresholds: 18.5, 24.9, 29.9, and 34.9. The left *y*-axis indicates the count of individuals in each BMI category, and the right *y*-axis shows the density of the BMI values. Figure S2. Selection of predictors for 365-day mortality using LASSO Cox regression. *Panel A* shows the results of the LASSO Cox regression for selecting predictors of 365-day mortality in patients on dual antiplatelet therapy (DAPT). The plot illustrates the cross-validation curve, where the *x*-axis represents the logarithm of the lambda values, and the *y*-axis represents the mean cross-validated partial likelihood deviance. The optimal lambda value, identified by the minimum mean cross-validated error, is used to select the most relevant predictors. *Panel B* presents the hazard ratios (HRs) for various predictors of 365-day mortality identified using LASSO Cox regression. The plot includes non-zero coefficients from the final model, with the variable names listed on the *y*-axis and their corresponding hazard ratios on the *x*-axis. Confidence intervals for the hazard ratios are represented by error bars. A positive HR indicates an increased risk of 365-day mortality, while a negative HR indicates a decreased risk. Figure S3. Survival data of patients treated with coronary intervention and dual antiplatelet therapy by WHO BMI categories. Kaplan–Meier curves showing 1-year survival by BMI categories after PCI. The curves demonstrate early and persistent separation across BMI strata, consistent with stable relative risks throughout follow-up. Table S1. Year-specific hazard ratios (HRs) for the association of BMI and P2Y12 inhibitor treatment with 1-year mortality (2014–2021). Models adjusted for the same covariates as in the main Cox analyses.

## References

[1] Valgimigli M., Bueno H., Byrne R.A., Collet J.P., Costa F., Jeppsson A., Jüni P., Kastrati A., Kolh P., Maur L. (2018). 2017 ESC focused update on dual antiplatelet therapy in coronary artery disease developed in collaboration with EACTS: The Task Force for dual antiplatelet therapy in coronary artery disease of the European Society of Cardiology (ESC) and of the European Association for Cardio-Thoracic Surgery (EACTS). Eur. Heart J..

[2] Angiolillo D.J., Saucedo J.F., DeRaad R., Frelinger A.L., Gurbel P.A., Costigan T.M., Jakubowski J.A., Ojeh C.K., Effron M.B., SWAP Investigators (2010). Increased Platelet Inhibition After Switching From Maintenance Clopidogrel to Prasugrel in Patients with Acute Coronary Syndromes: Results of the SWAP (SWitching Anti Platelet) Study. J. Am. Coll. Cardiol..

[3] Komócsi A., Merkely B., Hadamitzky M., Massberg S., Rizas K.D., Hein-Rothweiler R., Gross L., Trenk D., Sibbing D., Aradi D. (2023). Impact of body mass on P2Y12-inhibitor de-escalation in acute coronary syndromes—A substudy of the TROPICAL-ACS trial. Eur. Heart J. Cardiovasc. Pharmacother..

[4] Jakubowski J.A., Payne C.D., Brandt J.T., Weerakkody G.J., Farid N.A., Small D.S., Naganuma H., Li G.Y., Winters K.J. (2006). The Platelet Inhibitory Effects and Pharmacokinetics of Prasugrel After Administration of Loading and Maintenance Doses in Healthy Subjects. J. Cardiovasc. Pharmacol..

[5] Rocca B., Fox K.A.A., Ajjan R.A., Andreotti F., Baigent C., Collet J.-P., Grove E.L., Halvorsen S., Huber K., Morais J. (2018). Antithrombotic therapy and body mass: An expert position paper of the ESC Working Group on Thrombosis. Eur. Heart J..

[6] Wallentin L., Gale C.P., Maggioni A., Bardinet I., Casadei B. (2019). EuroHeart: European Unified Registries On Heart Care Evaluation and Randomized Trials. Eur. Heart J..

[7] Tornyos D., Lukács R., Jánosi A., Komócsi A. (2024). Prognosis Impact and Prediction of Trans-Radial Access Failure in Patients with STEMI, A Nationwide Observational Study. Am. J. Cardiol..

[8] Bálint A., Kupó P., Tornyos D., Abdallaoui O.E.A.E., Jánosi A., Komócsi A. (2021). Oral anticoagulation and outcomes in patients with acute myocardial infarction: Insights from the Hungarian Myocardial Infarction Registry. Int. J. Clin. Pract..

[9] Kupó P., Tornyos D., Bálint A., Lukács R., Jánosi A., Komócsi A. (2021). Use of drug-eluting stents in elderly patients with acute myocardial infarction: An analysis of the Hungarian Myocardial Infarction Registry. Int. J. Clin. Pract..

[10] Jánosi A., Ofner P., Forster T., Édes I., Tóth K., Merkely B. (2014). Clinical characteristics, hospital care, and prognosis of patients with ST elevation myocardial infarction: Hungarian Myocardial Infarction Registry. Eur. Heart J. Suppl..

[11] Jánosi A., Ferenci T., Komócsi A., Andréka P. (2021). A Kórelőzményben Szereplő Revascularisatiós Műtét Rövid és Hosszú Távú Prognosztikai Jelentősége Szívinfarktus Miatt Kezelt Betegekben. https://akjournals.com/view/journals/650/162/5/article-p177.xml.

[12] Vickers A.J., Elkin E.B. (2006). Decision curve analysis: A novel method for evaluating prediction models. Med. Decis. Mak. Int. J. Soc. Med. Decis. Mak..

[13] Wang Z.J., Gao F., Cheng W.J., Yang Q., Zhou Y.J. (2015). Body Mass Index and Repeat Revascularization After Percutaneous Coronary Intervention: A Meta-analysis. Can. J. Cardiol..

[14] Sharma A., Vallakati A., Einstein A.J., Lavie C.J., Arbab-Zadeh A., Lopez-Jimenez F., Mukherjee D., Lichstein E. (2014). Relationship of Body Mass Index with Total Mortality, Cardiovascular Mortality, and Myocardial Infarction After Coronary Revascularization: Evidence From a Meta-analysis. Mayo Clin. Proc..

[15] Kosuge M., Kimura K., Kojima S., Sakamoto T., Ishihara M., Asada Y., Tei C., Miyazaki S., Sonoda M., Tsuchihashi K. (2007). Impact of Body Mass Index on In-Hospital Outcomes After Percutaneous Coronary Intervention for ST Segment Elevation Acute Myocardial Infarction. Circ. J..

[16] Niedziela J., Hudzik B., Niedziela N., Gąsior M., Gierlotka M., Wasilewski J., Myrda K., Lekston A., Poloński L., Rozentryt P. (2014). The obesity paradox in acute coronary syndrome: A meta-analysis. Eur. J. Epidemiol..

[17] Romero-Corral A., Montori V.M., Somers V.K., Korinek J., Thomas R.J., Allison T.G., Mookadam F., Lopez-Jimenez F. (2006). Association of bodyweight with total mortality and with cardiovascular events in coronary artery disease: A systematic review of cohort studies. Lancet.

[18] Elagizi A., Kachur S., Lavie C.J., Carbone S., Pandey A., Ortega F.B., Milani R.V. (2018). An Overview and Update on Obesity and the Obesity Paradox in Cardiovascular Diseases. Prog. Cardiovasc. Dis..

[19] Lavie C.J., Milani R.V., Ventura H.O. (2009). Obesity and Cardiovascular Disease: Risk Factor, Paradox, and Impact of Weight Loss. J. Am. Coll. Cardiol..

[20] Ono M., Chichareon P., Tomaniak M., Kawashima H., Takahashi K., Kogame N., Modolo R., Hara H., Gao C., Wang R. (2020). The association of body mass index with long-term clinical outcomes after ticagrelor monotherapy following abbreviated dual antiplatelet therapy in patients undergoing percutaneous coronary intervention: A prespecified sub-analysis of the GLOBAL LEADERS Trial. Clin. Res. Cardiol..

[21] Wiviott S.D., Braunwald E., McCabe C.H., Montalescot G., Ruzyllo W., Gottlieb S., Neumann F.-J., Ardissino D., De Servi S., Murphy S.A. (2007). Prasugrel versus Clopidogrel in Patients with Acute Coronary Syndromes. N. Engl. J. Med..

[22] 2023 ESC Guidelines for the Management of Acute Coronary Syndromes. https://www.escardio.org/Guidelines/Clinical-Practice-Guidelines/Acute-Coronary-Syndromes-ACS-Guidelines#.

[23] Jakubowski J.A., Winters K.J., Naganuma H., Wallentin L. (2007). Prasugrel: A Novel Thienopyridine Antiplatelet Agent. A Review of Preclinical and Clinical Studies and the Mechanistic Basis for Its Distinct Antiplatelet Profile. Cardiovasc. Drug Rev..

[24] Wallentin L., Becker R.C., Budaj A., Cannon C.P., Emanuelsson H., Held C., Horrow J., Husted S., James S., Katus H. (2009). Ticagrelor versus Clopidogrel in Patients with Acute Coronary Syndromes. N. Engl. J. Med..

[25] Schüpke S., Neumann F.-J., Menichelli M., Mayer K., Bernlochner I., Wöhrle J., Richardt G., Liebetrau C., Witzenbichler B., Antoniucci D. (2019). Ticagrelor or Prasugrel in Patients with Acute Coronary Syndromes. N. Engl. J. Med..

[26] Öz A., Toprak K., Aydin E., Saraç İ., Doğduş M., Opan S., Yenerçağ M., Tascanov M.B., Kümet Ö., Karaağaç M. (2024). Fixed-Dose Antiplatelet Dual Combination in Patients with Coronary Artery Disease in Turkish Population: DAPT-TR. Arq. Bras. Cardiol..

[27] Lukács R.A., Tornyos D., Kupó P., Jánosi A., Komócsi A. (2024). The Comparative Effectiveness of Potent P2Y12 Inhibitors Versus Clopidogrel in Patients with Acute Myocardial Infarction Undergoing PCI: National Registry Data. J. Clin. Med..

[28] Collet J.P., Thiele H., Barbato E., Barthélémy O., Bauersachs J., Bhatt D.L., Dendale P., Dorobantu M., Edvardsen T., Folliguet T. (2021). 2020 ESC Guidelines for the management of acute coronary syndromes in patients presenting without persistent ST-segment elevation: The Task Force for the management of acute coronary syndromes in patients presenting without persistent ST-segment elevation of the European Society of Cardiology (ESC). Eur. Heart J..

[29] Rao S.V., O’donoghue M.L., Ruel M., Rab T., Tamis-Holland J.E., Alexander J.H., Baber U., Baker H., Cohen M.G., Cruz-Ruiz M. (2025). 2025 ACC/AHA/ACEP/NAEMSP/SCAI Guideline for the Management of Patients with Acute Coronary Syndromes: A Report of the American College of Cardiology/American Heart Association Joint Committee on Clinical Practice Guidelines. Circulation.

