# Supplementary material for: Impact of Body Mass Index on Clinical Outcomes in Myocardial Infarction Patients Undergoing Coronary Stenting with Dual Antiplatelet Therapy
Source: Biomedicines. 2025 Nov 16;13(11):2792. doi: 10.3390/biomedicines13112792 (PMC12650446; doi:10.3390/biomedicines13112792)
Supplement: Supplementary file 1 [file biomedicines-13-02792-s001.zip › biomedicines-3847317-supplementary.pdf]

Supplementary Material

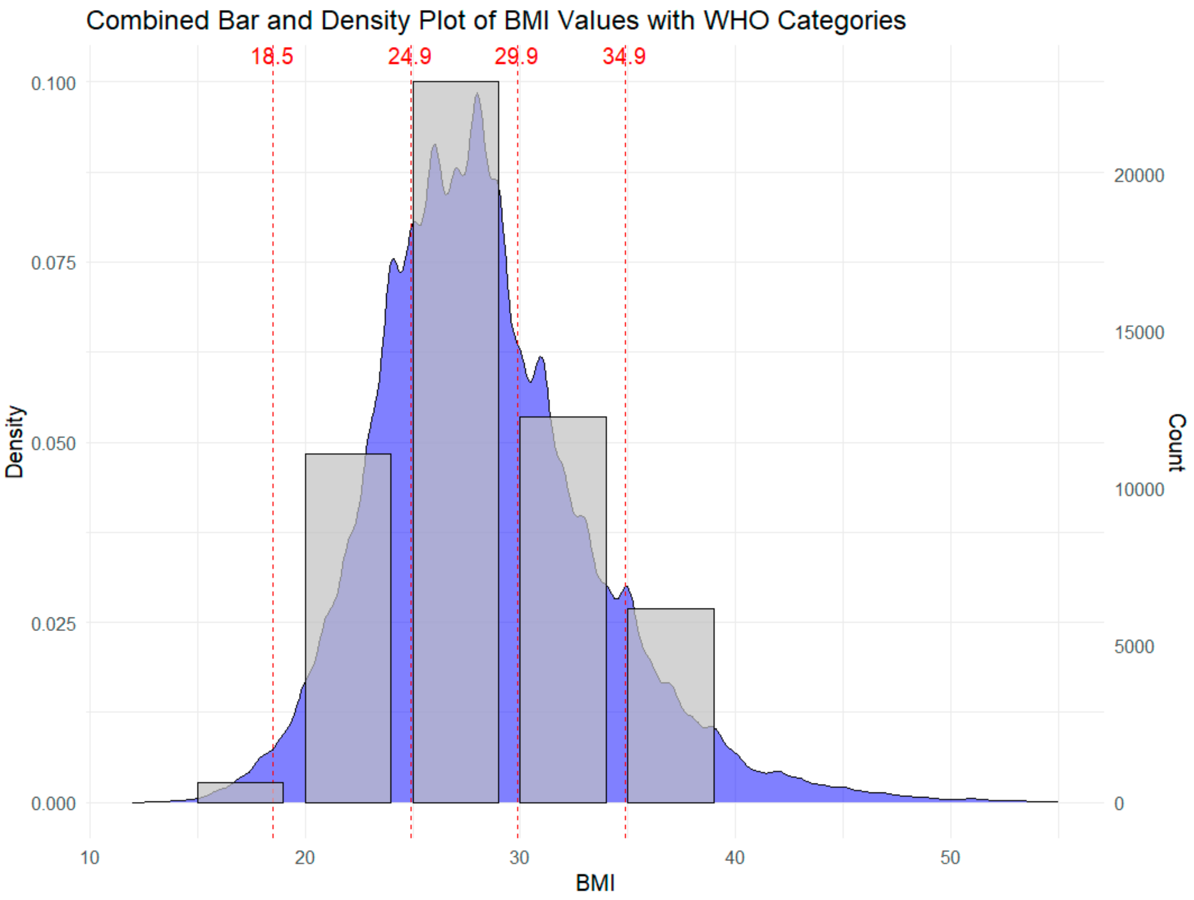

Supplementary Figure S1. Combined Density and Bar Plot of BMI Distribution with WHO Categories. This figure displays a combined density and bar plot illustrating the distribution of Body Mass Index (BMI) values among the study population. The density plot (in blue) represents the smooth distribution of BMI values, while the overlaid bar plot (in grey) shows the count of individuals within specific BMI categories. Vertical dashed red lines mark the World Health Organization (WHO) BMI classification thresholds: 18.5, 24.9, 29.9, and 34.9. The left y-axis indicates the count of individuals in each BMI category, and the right y-axis shows the density of the BMI values.

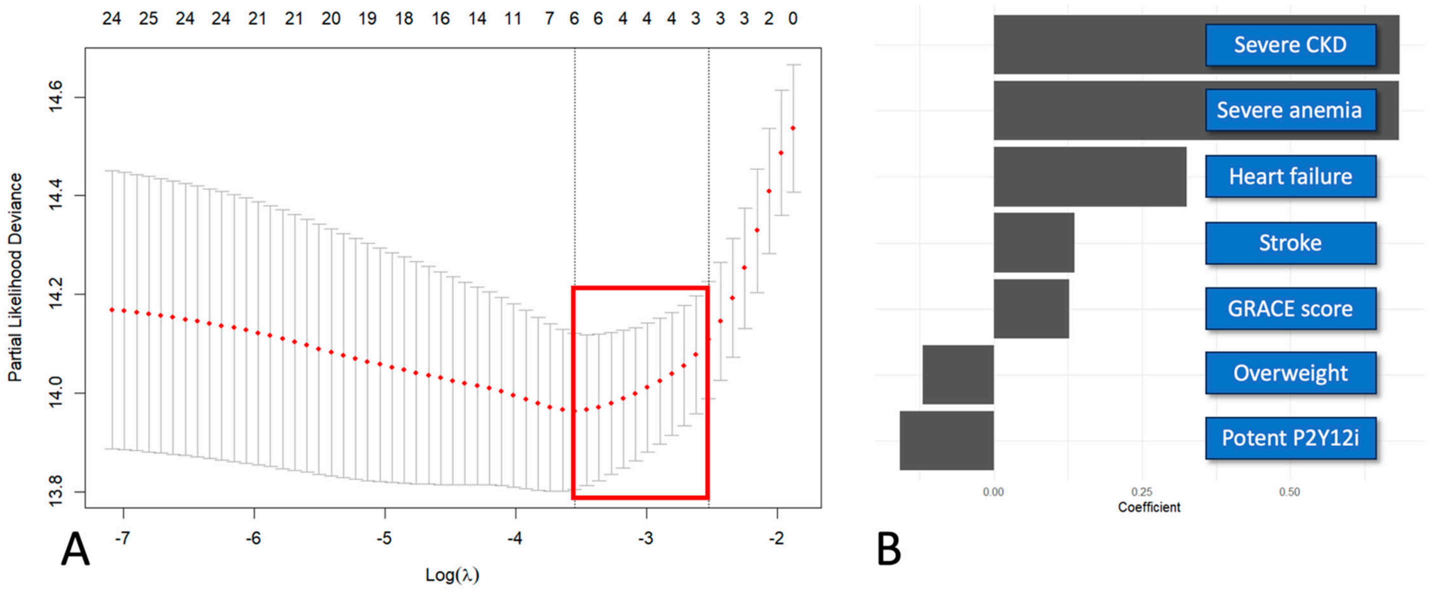

Supplementary Figure S2. Selection of Predictors for 365-Day Mortality Using LASSO Cox Regression. *Panel A* shows the results of the LASSO Cox regression for selecting predictors of 365-day mortality in patients on dual antiplatelet therapy (DAPT). The plot illustrates the cross-validation curve, where the x-axis represents the logarithm of the lambda values, and the y-axis represents the mean cross-validated partial likelihood deviance. The optimal lambda value, identified by the minimum mean cross-validated error, is used to select the most relevant predictors. *Panel B* presents the hazard ratios (HR) for various predictors of 365-day mortality identified using LASSO Cox regression. The plot includes non-zero coefficients from the final model, with the variable names listed on the y-axis and their corresponding hazard ratios on the x-axis. Confidence intervals for the hazard ratios are represented by error bars. A positive HR indicates an increased risk of 365-day mortality, while a negative HR indicates a decreased risk.

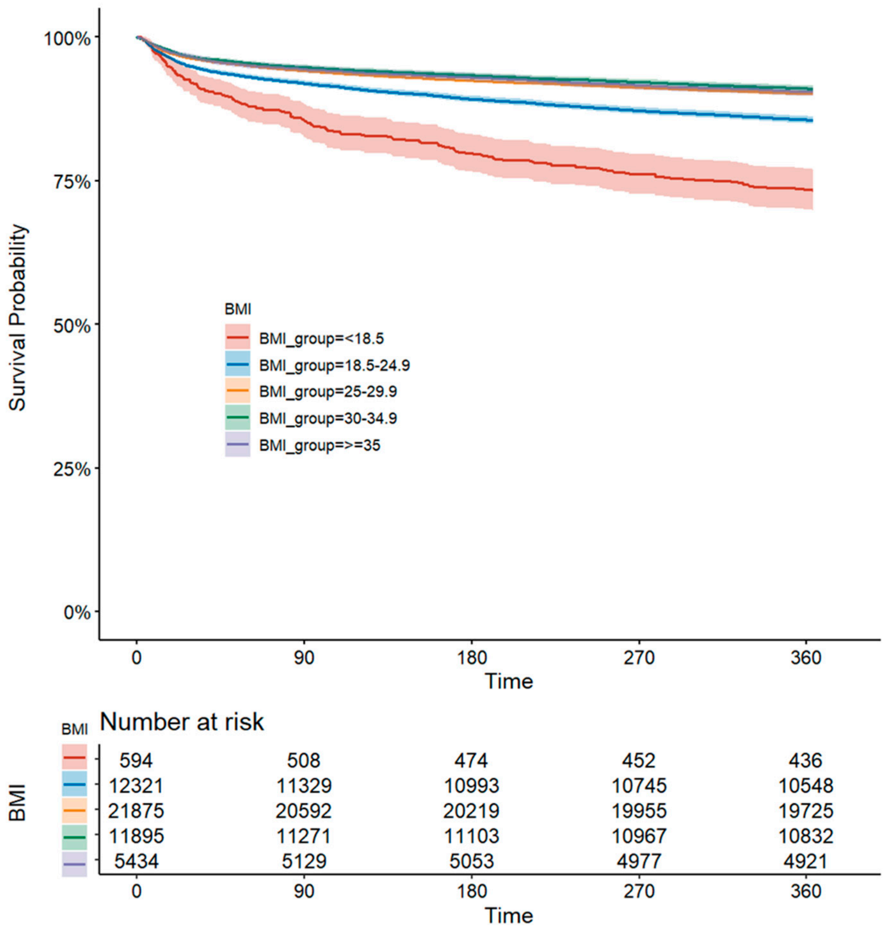

Supplementary Figure S3. Survival Data of Patients Treated with Coronary Intervention and Dual Antiplatelet Therapy by WHO BMI Categories. Kaplan–Meier curves showing 1-year survival by BMI categories after PCI. The curves demonstrate early and persistent separation across BMI strata, consistent with stable relative risks throughout follow-up.

Supplementary Table S1. Year-specific hazard ratios (HRs) for the association of BMI and P2Y12 inhibitor treatment with 1-year mortality (2014–2021). Models adjusted for the same covariates as in the main Cox analyses.

Year	Clopidogrel vs P2Y12i HR (95% CI)	BMI (per 5-unit increase) HR (95% CI)	p for interaction
2014	1.62 (1.12–2.32)	0.81 (0.73–0.88)	0.648
2015	3.35 (2.19–5.12)	0.83 (0.76–0.90)	0.126
2016	3.20 (2.05–4.99)	0.83 (0.77–0.90)	0.022
2017	2.60 (1.72–3.94)	0.78 (0.72–0.84)	0.976
2018	1.75 (1.26–2.43)	0.89 (0.83–0.96)	0.287
2019	1.89 (1.50–2.37)	0.95 (0.88–1.01)	0.103
2020	2.07 (1.70–2.51)	0.92 (0.86–0.99)	0.962
2021	1.43 (1.17–1.74)	0.81 (0.75–0.88)	0.137
